# Piano Fortes

**Published:** 1866-09

**Authors:** 


### PIANO FORTES.

THERE is a time-honored and mammoth building cornering on Fourteenth street and Third avenue, which has met the familiar gaze of such of our citizens as have been accustomed to pass that way, for perhaps a greater part of the present half century. This building stretching its immense length along two streets, is devoted exclusively to the manufacture of the Worcester Piano, which has a name for durability of structure and sweetness of tone which ought, if it has not, to have made the fortune of any man of moderate ambitions. But it is not as easy now as formerly, to make a fortune by strictly honest dealing; if done at all, it is only until a man has become decrepid and gray, and almost ready to take his departure on the returnless journey; one of the reasons of this is found in an article in the July number, headed "unskilled labor."

Another, at least temporary drawback, as to a speedy fortune by strict business integrity, is the want of means on the part of the many, to secure the best materials for their particular handicrafts. Sometimes on account of a want of foresight or thrift, or a still more unpardonable want of knowledge, materials are needed for the construction of a superior article, which no money can purchase, and time only can procure the needed supply.

Too many of our mechanical men live from hand to mouth, and the material purchased yesterday, must be used to-day; in proof, look at any floor in any brown stone or marbled front, in the whole city of New York, constructed within the last five years, and it will be scarcely possible to find a well-fitting door, an easy moving drawer or window sash, while the joints in the floors will measure from a quarter to half an inch or more. This is so undeniable, that builders find it the shortest cut to say, that it is owing to furnace heat; and yet, Forty-two Irving Place, which has a furnace only for appearances, can show floors on either story half an inch apart at the ends of the boards, and at the sides in proportion.

When, however, there is a business integrity, and abundant means to employ the best materials in fabrics of any description, two results always show themselves, a good name and an ultimate prosperity; hence the reputation and success of the establishment in question, whose instruments stand the test of all weathers, from Canada to Cuba, and from the borders of the Atlantic to the shores of the Pacific Sea.

To make this practically useful to all young mechanics, the secret should be communicated, and it consists in three things:

1. A faithful apprenticeship to a good master.
2. A timely supply of the very best materials;
3. Making them up without haste, and with the utmost carefulness.

In the case above, the wood of important parts is obtained years beforehand; it undergoes a most minute examination as to its soundness, passing through a long seasoning, according to the varying thickness and hardness of the particular wood, and if at the end of this tedious process, the material remains sound and hard, without a blemish, it is used, and not otherwise. It is thus by making each particular instrument as if for his own personal use, almost living in the same building with the workmen, passing through every room at any hour of the day, making the employés feel as if they were watched every moment; it is by these means, we repeat, that the Piano Fortes of this house have acquired a reputation at home and abroad, which requires an almost daily shipment to other countries as well as to the various parts of our own.

To every young mechanic we therefore say, the path of a certain and honorable success for you is,

1. Be thorough masters of your calling; and,
2. Give honest material and honest work to every article which leaves your establishment.

